# Supplementary material for: Designing New Sustainable Polyurethane Adhesives: Influence of the Nature and Content of Diels–Alder Adducts on Their Thermoreversible Behavior
Source: Polymers (Basel). 2022 Aug 19;14(16):3402. doi: 10.3390/polym14163402 (PMC9414518; doi:10.3390/polym14163402)
Supplement: Supplementary file 1 [file polymers-14-03402-s001.zip › polymers-1754174-supplementary.pdf]

polymers

Supplementary Materials: Designing New Sustainable Polyurethane Adhesives: Influence of the Nature and Content of Diels–Alder Adducts on their Thermoreversible Behavior

Susana Quiles-Díaz, Helga Seyler, Gary J. Ellis, Peter S. Shuttleworth, Araceli Flores, Marián A. Gómez-Fatou and Horacio J. Salavagione *

1. Characterization of thermoreversible DA molecules.

^{1}HNMR and ^{13}CNMR were used to confirm the synthesis of **DAF** and **DAR**. **Figures S1, S2** and **S3** show the ^{1}H NMR spectra of N,N′-(1,3-phenylene)bismaleimide (BMI), furfuryl alcohol (FA) and the **DAR** product, respectively. The ^{1}H NMR spectrum of **BMIR** displays a sharp signal at 6.84 ppm (intensity = 4H) corresponding to the hydrogen atoms linked to the alkene carbons (positions 10, 11, 14 and 15 in the inset structure) and a series of signals in the range of 7.35- 7.60 ppm due to protons in the aromatic ring (at positions 2, 3, 4 and 6) (**Figure S1**).

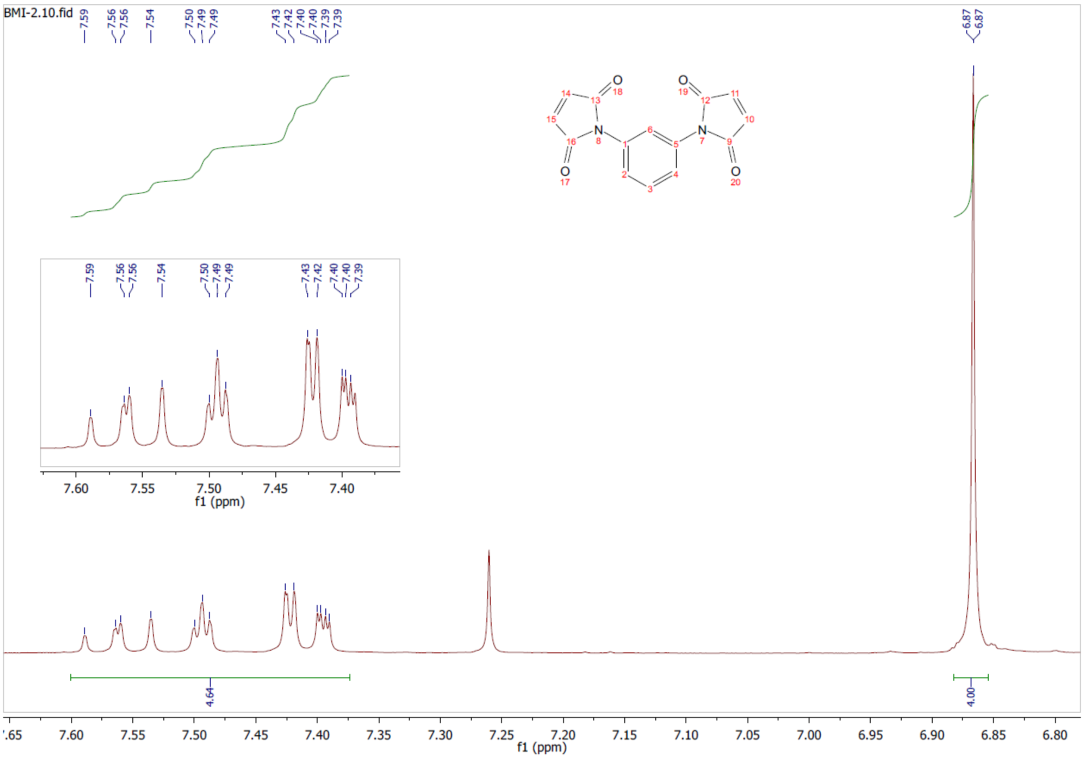

Figure S1. ^{1}H NMR spectrum of N,N′-(1,3-phenylene)bismaleimide in CHCl3-d.

The spectrum of furfuryl alcohol (**Figure S2**) shows the following signals: a singlet at 1.85 ppm (intensity = 1 H) assigned to the proton in the alcohol group (position 7); a doublet at 4.61 ppm (intensity = 2H) corresponding to the methylene protons (position 6); two multiplets at 6.34 ppm (intensity = 1H) and 6.30 ppm (intensity = 1H) corresponding to protons at position 2 and 3, respectively, and 7.74 ppm (intensity = 1H) due to the proton at position 1.

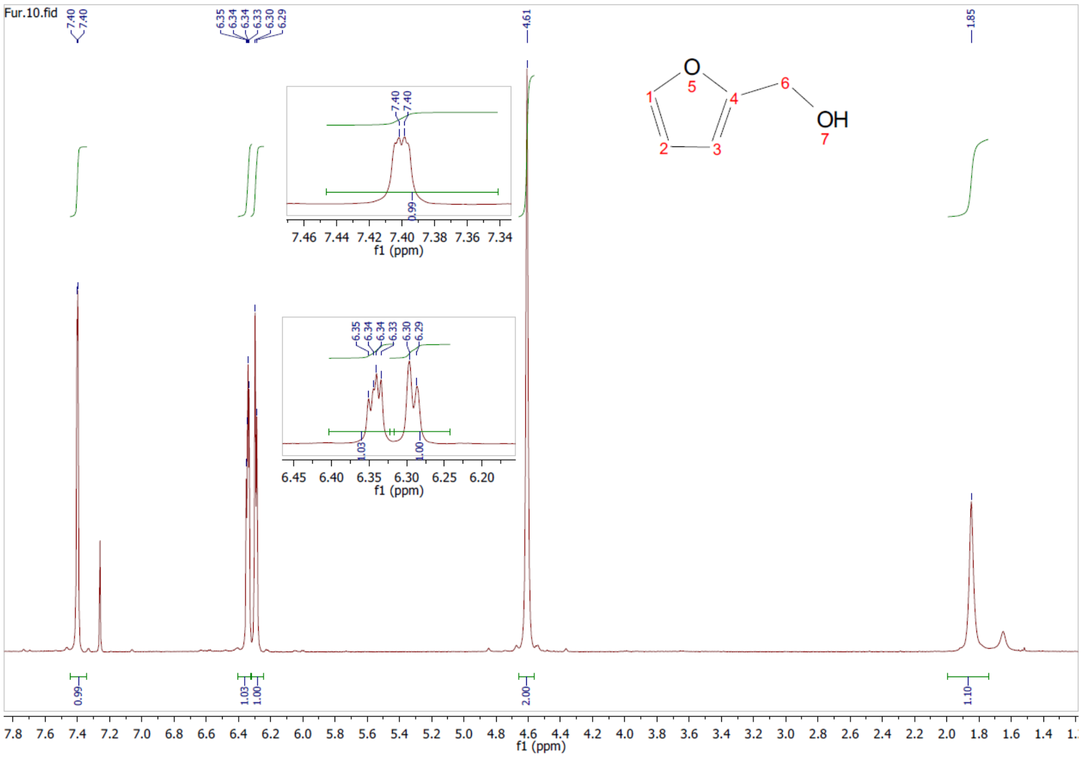

Figure S2. ^{1}H NMR spectrum of furfuryl alcohol, collected in CHCl3-d.

The spectrum of the **DAR** product presents several signals, each of which can be assigned to a specific proton in the molecule (**Figure S3**). As can be seen, all proton signals can be discerned and they fully agree with the data in the literature for similar compounds [1,1,2]. It is important to note that the coupling of furan derivatives and maleimides leads to a product with a mixture of stereoisomers, i.e., the endo- and exo-adducts, normally with an excess of the endo-adduct. This is also reflected in **Fig S3**, where the signals for protons (d), (e), (g) and (h) for endo- and exo-isomers are clearly observed. All these signals provide evidence for the formation of the desired DAR molecule. In addition, there is a complete absence of any band arising from **BMIR**, which implies that it has been fully consumed in the synthesis of DA under the conditions detailed in the main manuscript. On the other hand, the bands corresponding to furfuryl alcohol are clearly observed as expected, in principle, considering the large excess of FA in the feed.

Figure S4 shows the ^{13}C NMR of **DAR**. The 13C NMR spectrum of the **DAR** is somewhat more complex since, as evidenced by ^{1}H NMR, it presents a mixture of both isomers. However, all carbon signals are discerned and assigned to a specific position in the molecular structure of **DAR** in good agreement with previous reports on similar compounds [3]. The presence of signals other than those corresponding to the **DAR** molecule is also observed. These five additional signals, marked with an asterisk in **Figure S4**, correspond to residual FA occluded into the product.

1. [1] C. Jegat, N. Mignard. Effect of the polymer matrix on the thermal behavior of a furan-maleimide type adduct in the molten state. Polym. Bulletin 2008, 60, 799-808.

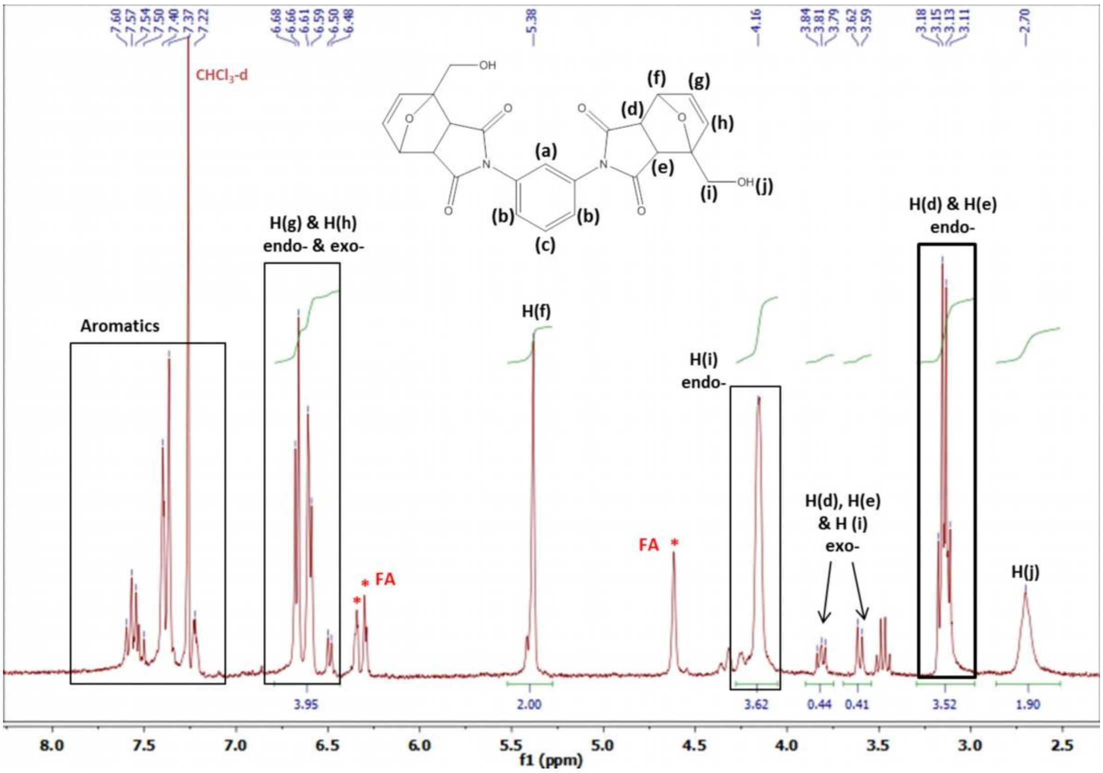

Figure S3. ^{1}H NMR spectrum of the DAR product, collected in CHCl3-d.

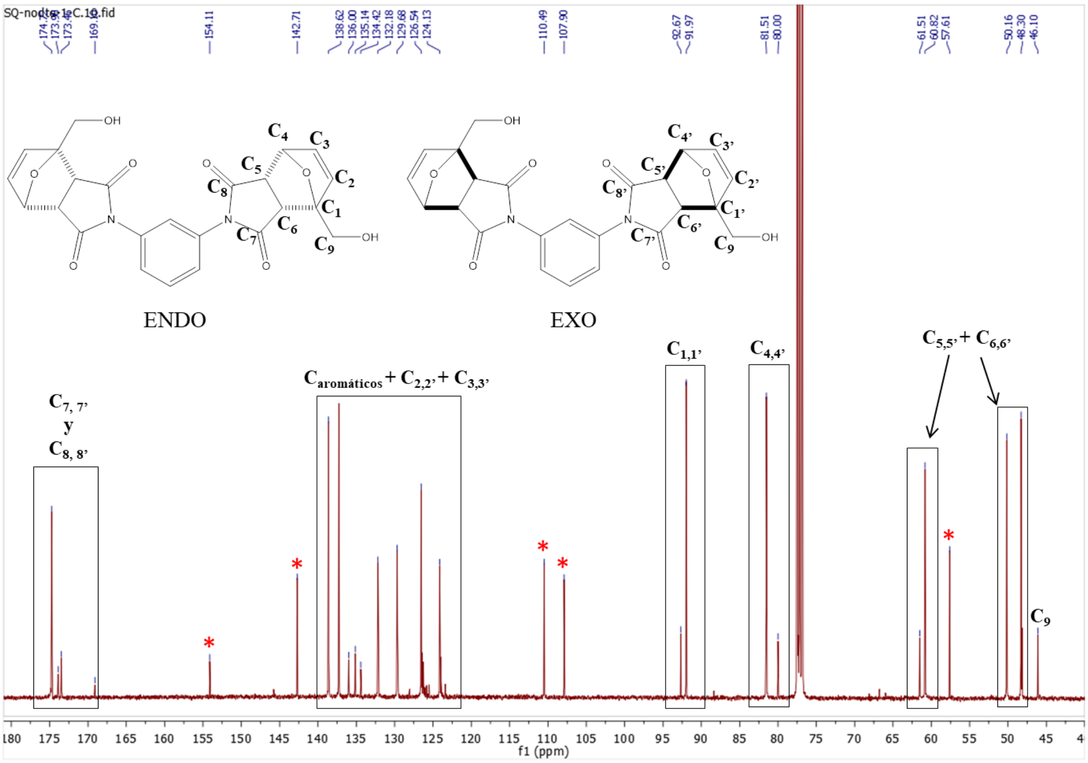

Figure S4. ^{13}C NMR spectrum of the DAR product.

Figure S5 and **S6** show the ^{1}HNMR spectra of 1,1′-(methylenedi-4,1-phenylene)bismaleimide (**BMIF**) and the **DAF** product, respectively. The ^{1}HNMR spectrum of 1,1′-(methylenedi-4,1-phenylene)bismaleimide (BMIF) (**Figure S5**) displays a signal at 4.09 ppm (intensity = 2H) corresponding to hydrogen atoms of the methylene group linking both aromatic rings (protons in position 1 in the inset structure); a sharp signal at 7.00

ppm (intensity = 4H) corresponds to hydrogen atoms linked to the alkene carbons (hydrogens 6 and 7) and a series of signals in the range of 7.20- 7.40 ppm due to protons in the aromatic rings (protons at positions 2, 3, 4 and 5).

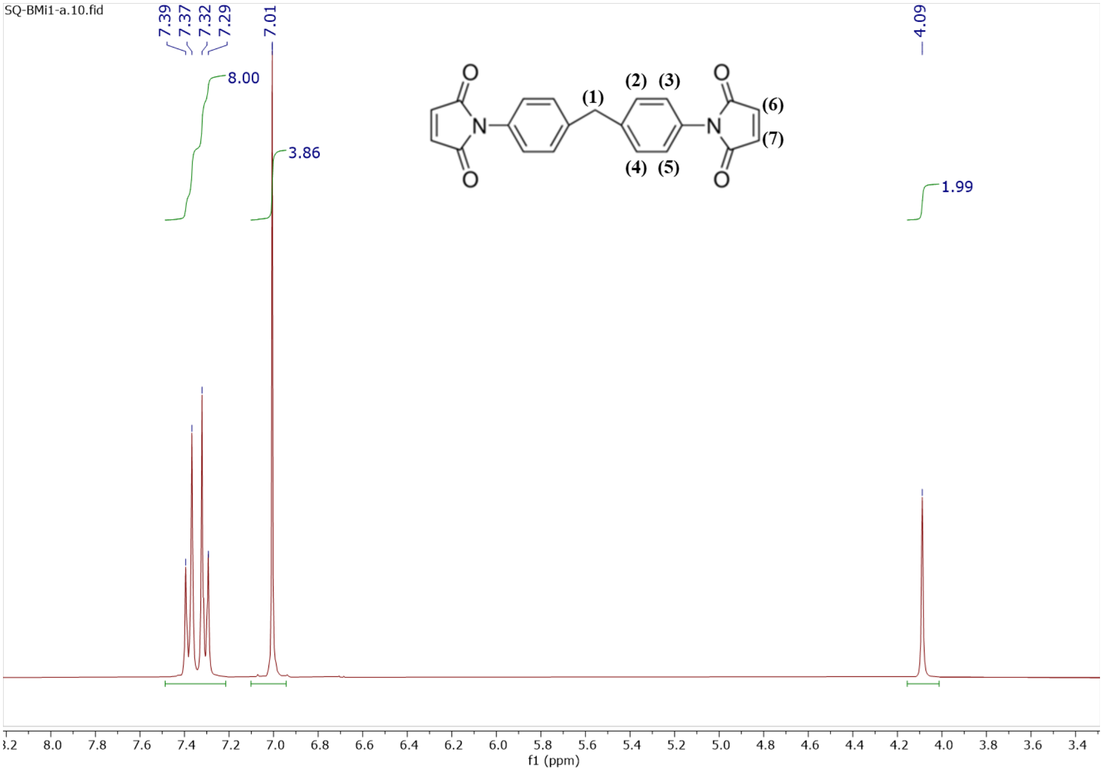

Figure S5. ^{1}HNMR spectrum of 1,1′-(methylenedi-4,1-phenylene)bismaleimide (BMIF) in deuterated acetone.

The spectrum of the **DAF** product presents several signals, each of which can be assigned to a specific proton in the molecule. As can be seen in **Figure S6**, all proton signals can be discerned and they fully agree with the data in the literature [1-3]. Therefore, the signals are in good agreement with the chemical structure of **DAF**. A mixture of signals coming from the endo- and exo-stereoisomers are obtained (as is also the case for **DAR**), as seen in the (g), (h) and (f) proton signals. Moreover, some additional signals that are not attributed to the **DAF** molecule appear in the ^{1}HNMR spectra, and have been marked with an asterisk in the figure corresponding to residual FA in the reaction product. In addition, there is a complete absence of any band arising from BMI, which implies that it has been fully consumed in the synthesis of DA under the conditions detailed in the manuscript.

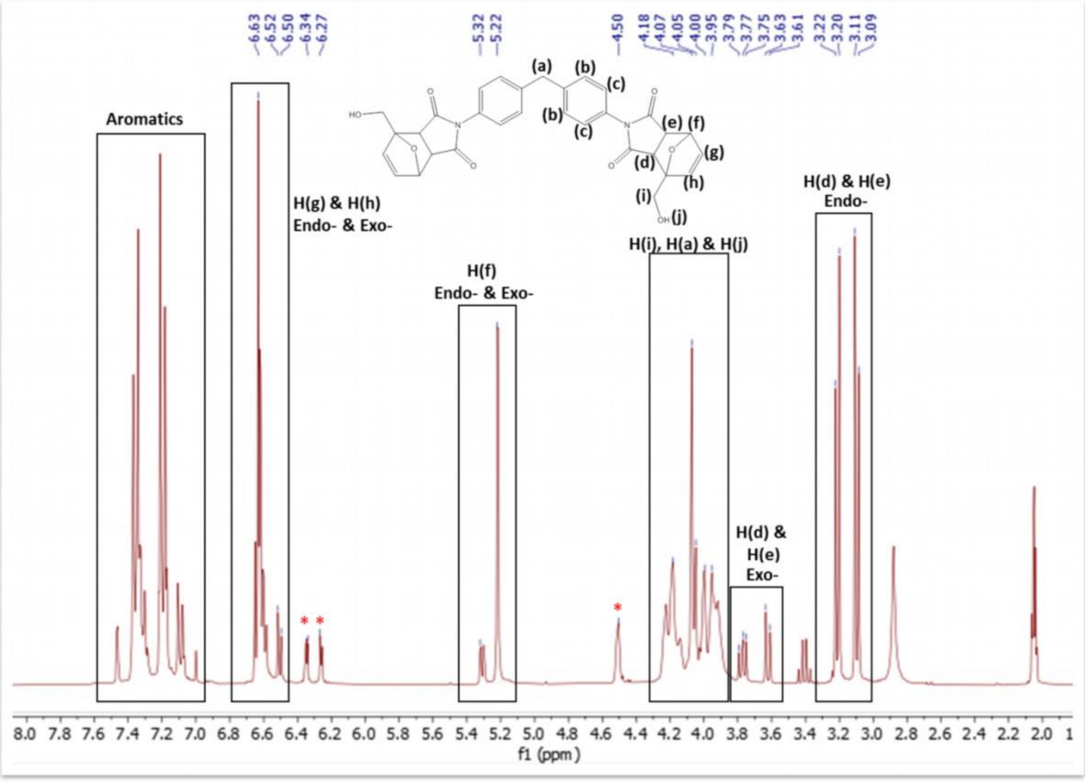

Figure S6. ^{1}HNMR spectrum of DAF in deuterated acetone.

.

Figures S7 and **S8** show the ^{13}CNMR spectra of the **BMIF** and **DAF**, respectively. Both compounds clearly show the signals arising from each carbon as denoted in the structure inserted in the figures.

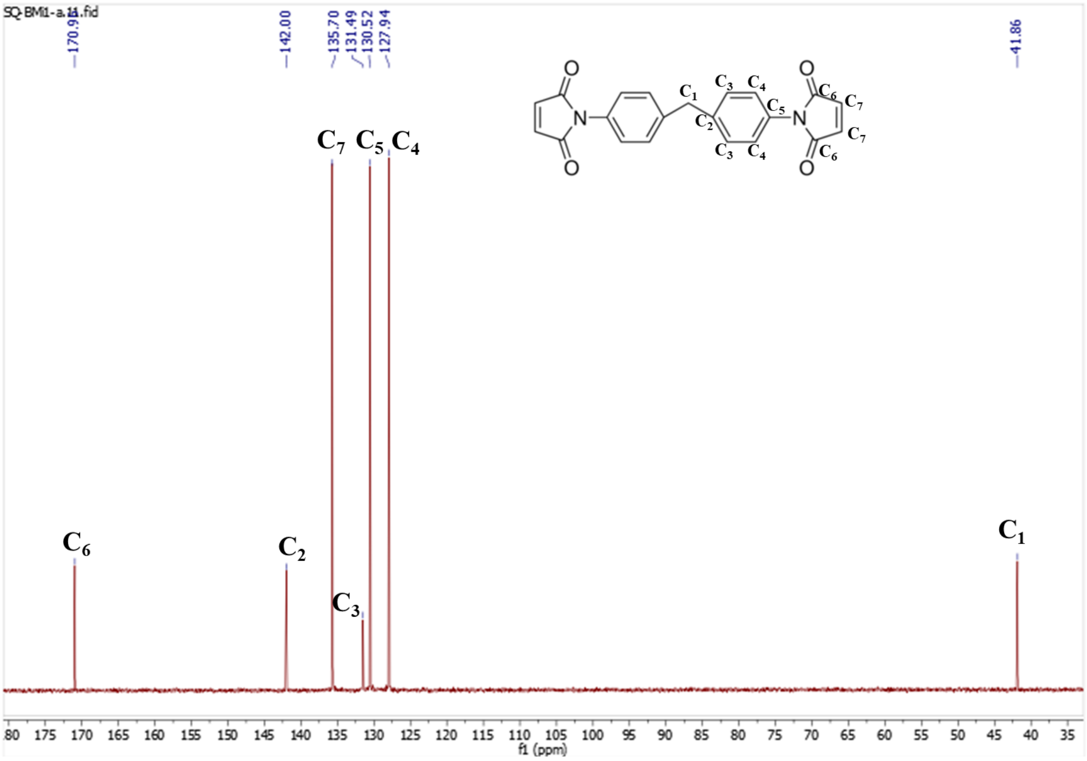

Figure S7. ^{13}CNMR spectrum of 1,1′-(methylenedi-4,1-phenylene)bismaleimide (BMIF) in deuterated acetone..

In accordance with the previous observations by ¹HNMR, the ¹³CNMR spectrum of **DAF** (**Figure S8**) shows the signals of both endo- and exo-stereoisomers. Signals ascribed to the presence of residual FA are also observed and are marked with an asterisk in the figure. In conclusion, proton and carbon NMR results are highly consistent and confirm the successful synthesis of **DAF**.

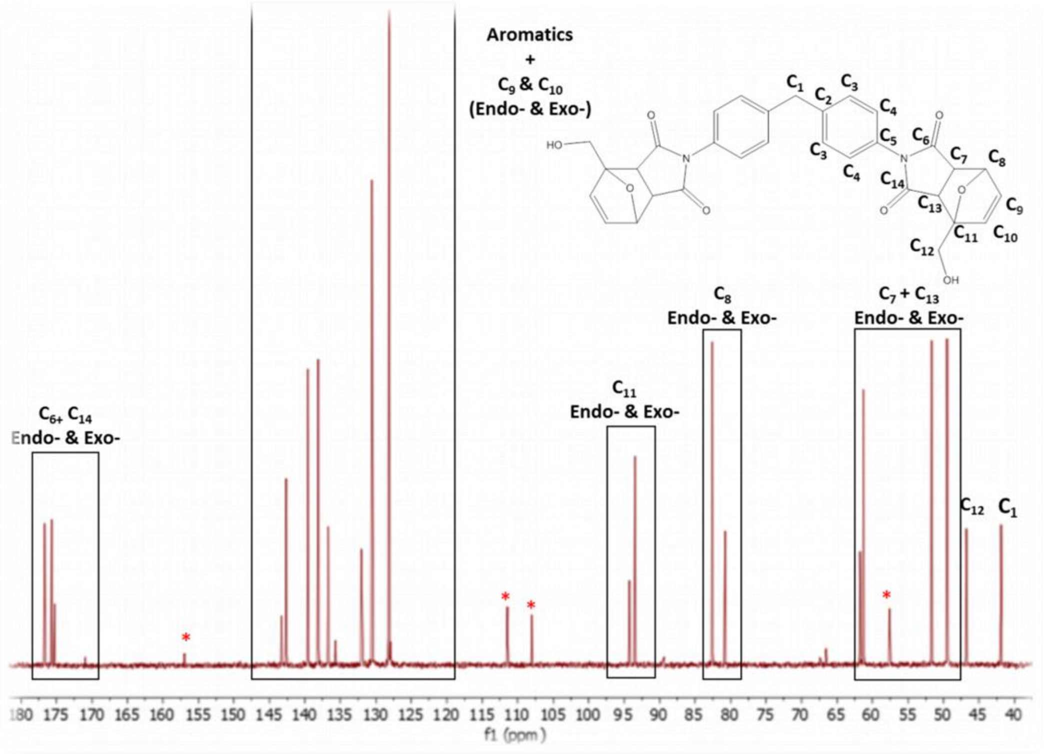

Figure S8. ¹³CNMR spectrum of the DAF product in deuterated acetone.

It is well established that the retro Diels–Alder (rDA) reaction, a process in which DA molecules revert back to the starting reagents, requires high temperatures, usually in the range of 90-130ºC. The rDA reaction was monitored by UV–Vis spectroscopy by following the appearance of the BMI band during the thermal treatment. A calibration curve was constructed in order to evaluate the sensitivity of this band. Accordingly, several solutions with an increasing concentration of 'flexible' BMI (BMIF) were prepared in 1,4-dioxane and the evolution of their UV–Vis spectra is shown in **Figure S9A**. Subsequently, the calibration curve was constructed by measuring the absorption of the different solutions at a wavelength of 350 nm and is represented in **Figure S9B**. This wavenumber was selected taking into consideration that the DA molecules present some spectral contribution in the region of the maximum of the BMI band. Hence, in order to differentiate the BMI coming from the rDA reaction, a wavelength slightly higher than the peak maximum was selected.

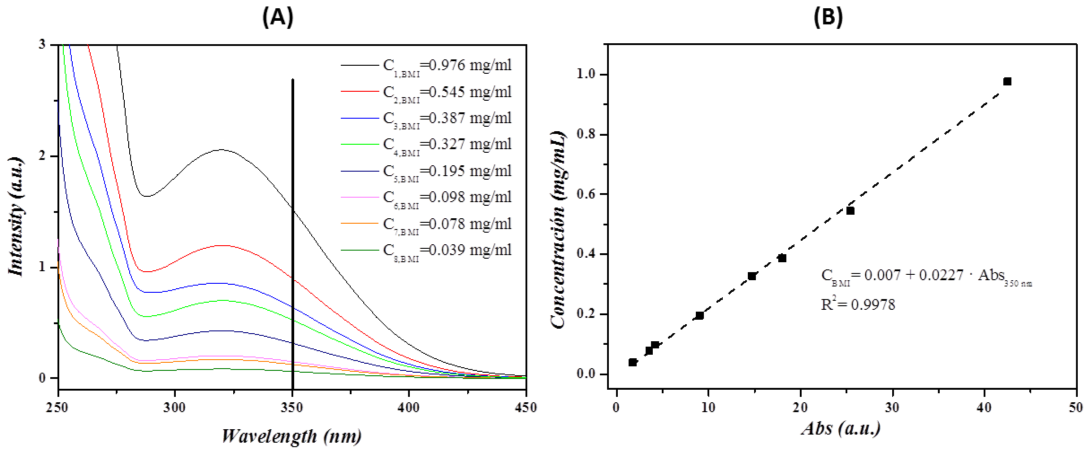

Figure S9. (**A**) UV–Visible spectra of different concentrations of BMIF in dioxane and (**B**) calibration curve for the quantification of the flexible BMIF concentration in solution with UV–Vis spectroscopy.

The evolution of the **DAF** or **DAR** absorption spectra with temperature is shown in **Figure S10**. The samples were heated to the target temperature using a ramp of 5 ºC·min^{-1} and t = 0 was considered as the time when this temperature was reached. The selected target temperatures were 70 °C and 90 °C. In all cases, a new band appearing at 310 nm or 320 nm which corresponded to the BMIF or BMIR, respectively, formed during the thermal treatment [4,5,6]. From **Figure S10**, it is clear that the BMI band appeared for both adducts and at both temperatures, reaching higher intensities at 90°C, regardless of the DA adduct evaluated. A comparison of both samples, **DAF** and **DAR**, at a particular time shows that the absorption was much higher for **DAF** when the sample was annealed at 70°C. In contrast, at 90°C, higher absorption values were reached for **DAR**. Differences in absorbance are directly related to changes in the amounts of BMI produced by the rDA reaction.

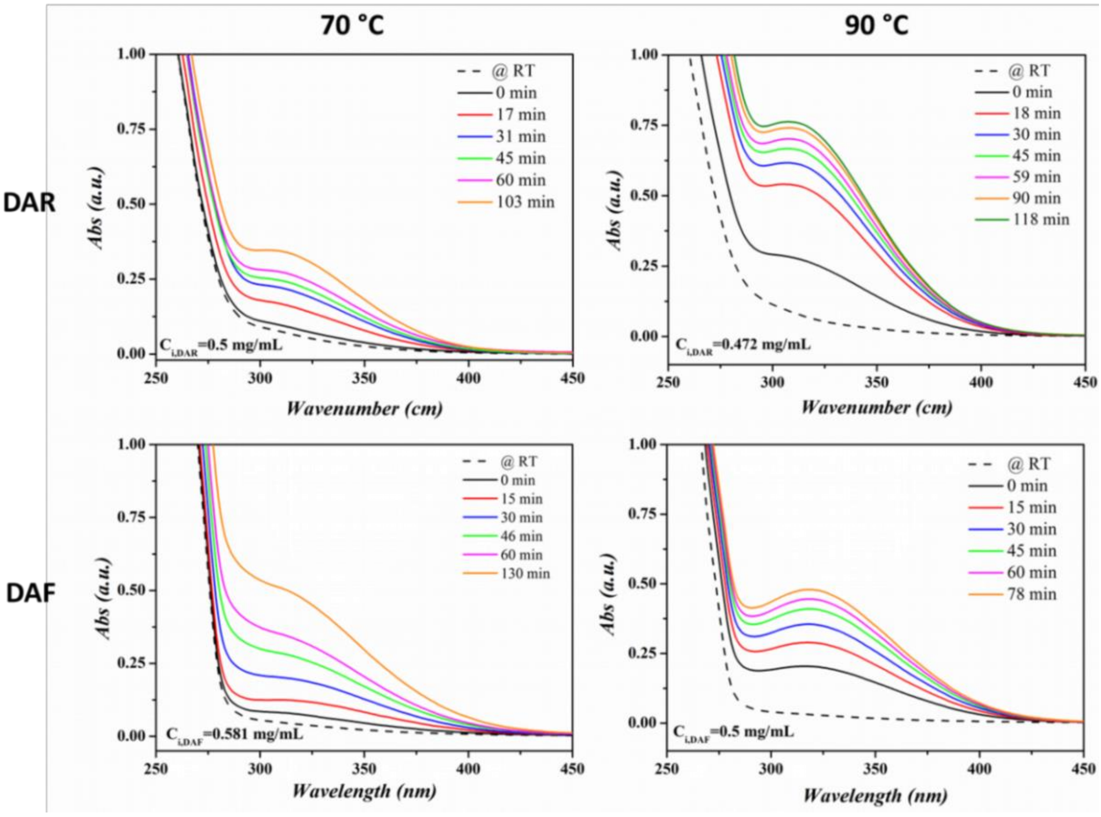

Figure S10. Time dependence of UV–Visible spectra of DAR and DAF solutions in dioxane at two different temperatures: A) 70ºC; B) 90ºC.

In order to further analyse the influence of the nature of the DA adducts on the efficiency of the rDA reaction, the degree of conversion was estimated using previously constructed BMI calibration curves (**Figure S9**). **Figure S11** compares the evolution of the rDA reaction degree with time for both types of DA adducts at the two temperatures.

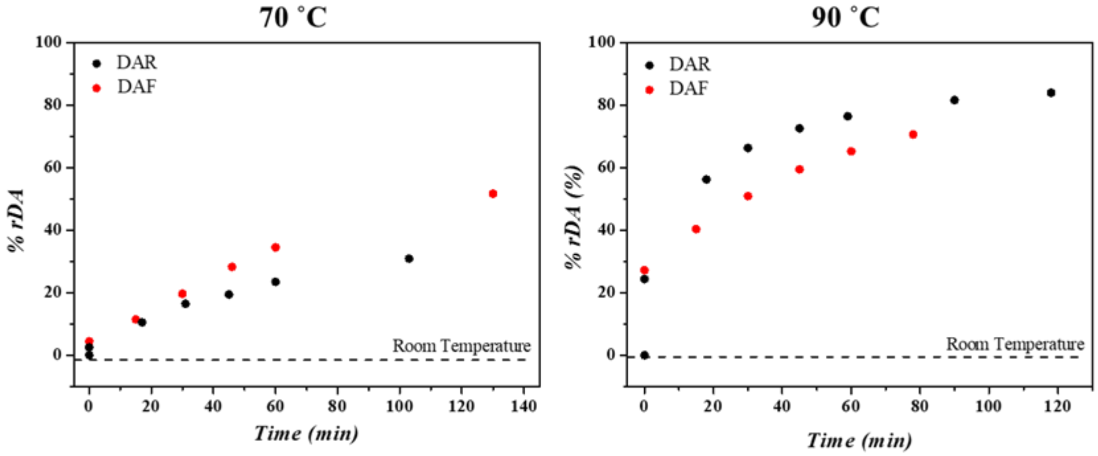

Figure S11. Variation in the degree of rDA conversion with time for DAR and DAF adducts at two temperatures.

From the results shown in **Figure S11**, a comparison of the thermoreversibility of the rigid and the flexible DA molecules can be established. **DAR** requires higher temperatures than **DAF** for the rDA reaction to become dominant. Regardless the type of adduct, at a given time higher rDA reaction yields are achieved at 90 °C than at 70 °C. For instance, by heating **DAF** solution at 70 °C for 60 min a 34% rDA is obtained, whereas a 65% rDA is achieved if it is maintained at 90 °C. Moreover, the results show that once a temperature threshold has been surpassed, in this case at T > 70 °C, **DAR** is more sensitive to the thermal treatment than **DAF**, since higher degrees of rDA reaction are obtained for **DAR** when the sample is heated at 90 °C.

2. Incorporation of thermoreversible DA molecules into solvent-based PUR adhesives.

The different solvent-based adhesives prepared using **DAF** and **DAR** adduct are given in **Table 2**. PUR-B01 corresponds to the blank sample, with neither DA nor FA molecules. A set of samples with 60 % OH content coming from the polyol and the remaining 40 % from DA adduct or its combination with FA (PUR-B04, PUR-B05, PUR-B11 and PUR-B12 in **Table 2**) were prepared and photographs of the films are shown in **Figure S12**. A similar behaviour to that described in the main manuscript for samples with 70 % OH content was observed for films containing **DAF**. However, for films incorporating **DAR**, its dispersion was less effective in absence of FA, as it contained poorly dispersed solid particles distributed over the entire film (bottom row in **Figure S12**). The use of FA significantly improved the incorporation of **DAR**, although a few agglomerated particles could be seen. Therefore, it can be assumed that a minimum of 10 % OH equivalents coming from the FA is required to homogeneously disperse the 30 % OH eq. from the **DAR** (20% of thermoreversible bonds in the system). Nevertheless, any further increase in the FA content will compromise the concentration of the number of thermoreversible bonds present within the system.

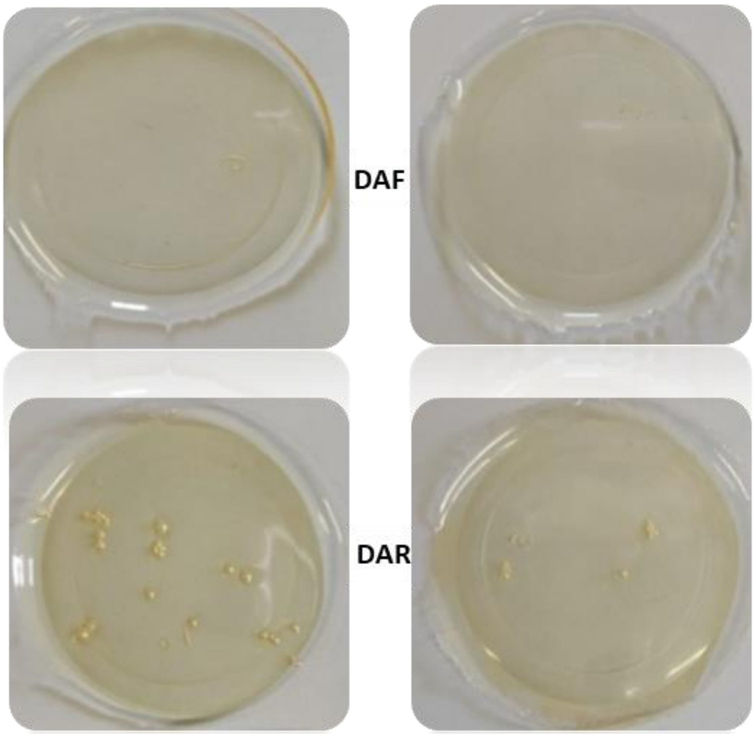

Figure S12. Comparison of the adhesive films with 60 % –OH groups from polyol incorporating DAF without FA (top left: PUR-B04) and with FA (top right PUR-B05) or DAR (bottom left: PUR-B11; bottom right PUR-B12)..

Another set of samples with only 50% of OH equivalents from polyol were then prepared, with the remaining 50% coming from the DA adducts or their combination with FA (PUR-B06 to PUR-B08 and PUR-B13 to PUR-B17 in **Table 2**). This high DA/FA ratio allows us to optimize the amounts of FA required to improve the dispersion of the **DAR** adduct, while maintaining the concentration of thermoreversible bonds above the critical value. Hence, in this case it is possible to tailor the DA/FA ratio to prepare a greater number of samples. The results are shown in **Figure S13** and **S14** for **DAF** and **DAR**, respectively. In the case of **DAF**, three different compositions were evaluated and in all cases the films were free of solid particles (**Figure S13**). It is interesting to highlight the particularity of the sample PUR-B06, containing 50% of OH from **DAF**, since it provided the highest amount of thermoreversible bonds of all the samples studied and produced homogeneous, transparent and flexible films. However, such a low amount of polyol could alter the adhesion properties of the product.

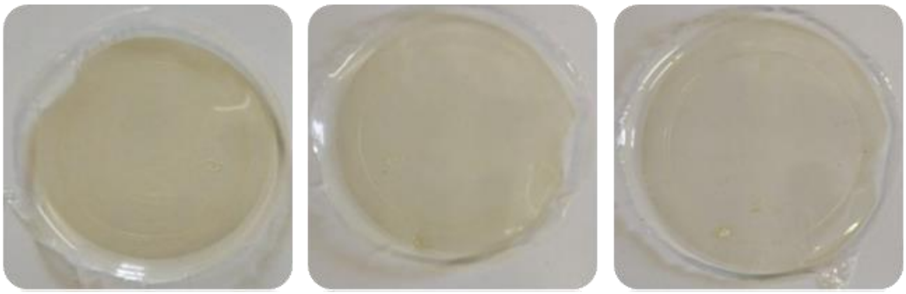

Figure S13. Comparison of the adhesive films with 50 % of –OH groups from polyol incorporating DAF without FA (PUR-B06) and with FA (PUR-B07 and PUR-B08).

In the case of **DAR**, five samples with different compositions were prepared and their films are shown in **Figure S14**. The DA/FA ratios employed were: 50%/0 (PUR-B13); 40/10 (PUR-B14); 35/15(PUR-B15); 30/20 (PUR-B16) and 25/25 (PUR-B17). It can be observed that

the sample without FA contained impurities, but these diminished as the FA content increased. From this set of samples, it can be assumed that at least 20 % of OH groups from FA are required to achieve homogeneous films. A sample with 20 % of FA (PUR-B16) contains around 20% of thermoreversible bonds, which meets the requirements to trigger the rDA reaction with noticeable effects on chain mobility at high temperature. However, as in the case of **DAF**, it is important to be sure that low amounts of polyol are not detrimental for the properties of the system.

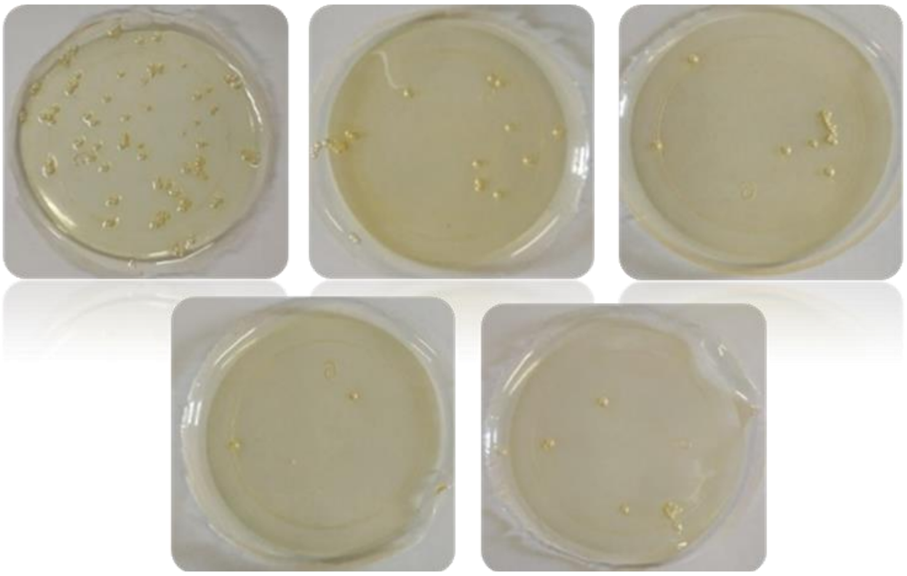

Figure S14. Comparison of the adhesive films with 50 % of –OH groups from polyol incorporating DAR without FA (PUR-B13) and with FA. The content of FA increases from left to right and from top to bottom (PUR-B14 to PUR-B17).

The thermal stability analysis of PUR with different amounts of DAR is shown in **Figure S15**.

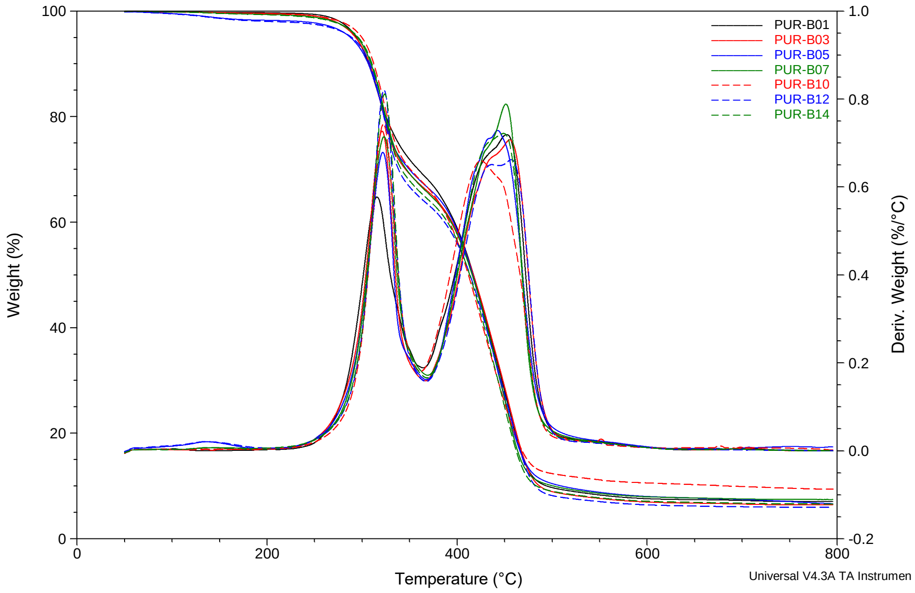

Figure S15. TG and DTG curves under a nitrogen atmosphere at a rate of 10ºC min-1 for the solvent-based adhesive (PUR-B01) and the solvent-based adhesives with **DAF** (PUR-B03, PUR-B05, PUR-B07) and with **DAR** (PUR-B-10, PUR-B12, PUR-B-14).

The variation in storage modulus from nanoindentation tests on some representative adhesives of this study is shown in **Figure S16.**

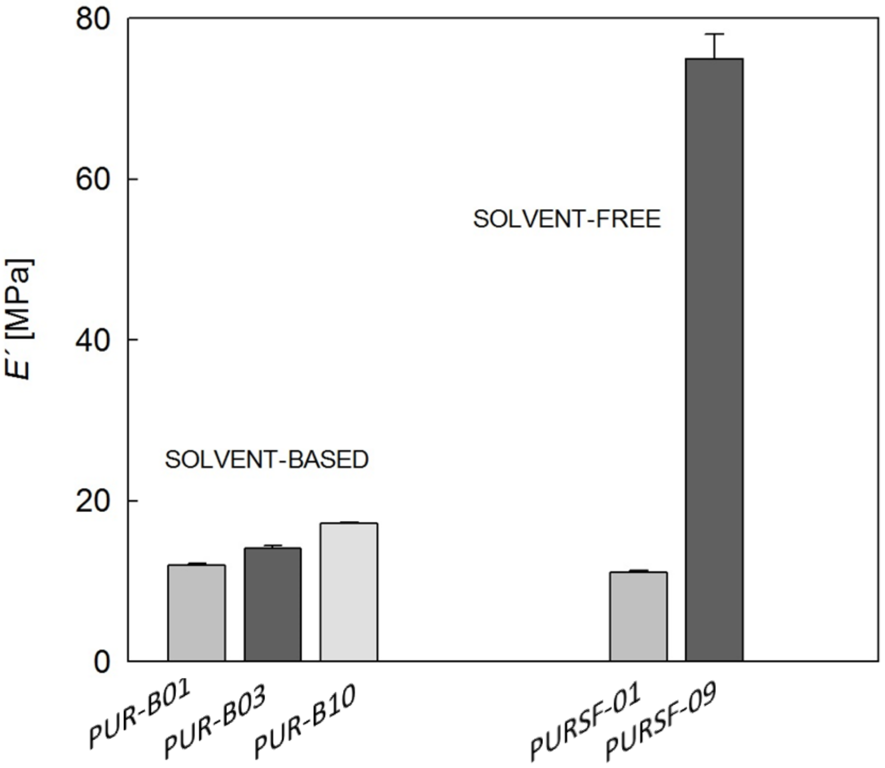

Figure S16. Variation in the storage modulus for some representative samples of both solvent-based and solvent-free adhesives.

3. Incorporation of thermoreversible DA molecules into solvent-free PUR adhesives.

The different solvent-free adhesives prepared using the **DAF** adduct are listed in **Table 3.** PURSF-01 corresponds to the blank sample without **DAF** and PURSF-02 to the blank sample with FA.

Photographs of different adhesive films taken after curing are shown in **Figure S17**, where it can be clearly observed that **DAF** was successfully dispersed in the system. Up to a content of 12 wt.% of thermoreversible bonds, the films with **DAF**/FA appeared transparent without the presence of any solid particles or bubbles regardless of the use of FA. In the absence of FA, **DAF** was incorporated into the system obtaining homogeneous and optically transparent films up to 15% of the thermoreversible bonds. The use of FA in this system did not appear to have the same effect as in the solvent-based system. The images in the bottom row of **Figure S17** suggest that lower amounts of **DAF** can be homogeneously incorporated when FA is used, since the sample with 15% of thermoreversible bonds presents some solid particles distributed within the film. These results indicate that the use of FA does not assist the incorporation of DAF in the solvent-free adhesive system, contrary to what occurred with the solvent-based system.

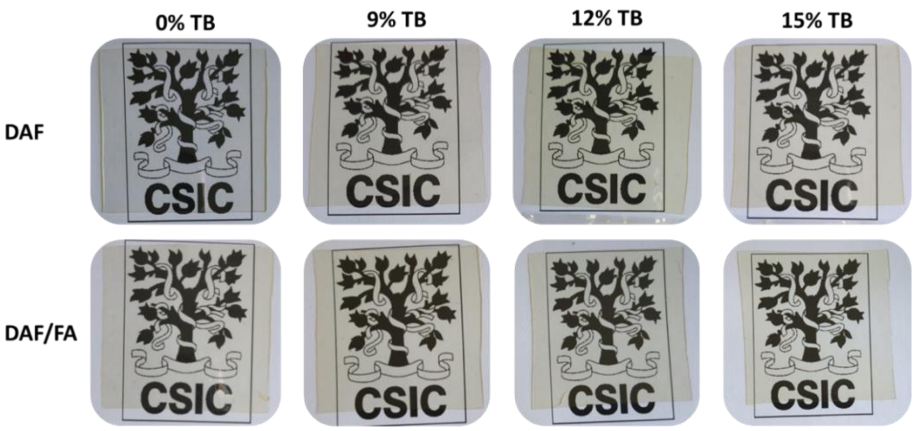

Figure S17. Comparison of the adhesive films with increasing DAF content from left to right. Top row: samples prepared without FA; bottom row: samples prepared with FA.

The thermal stability analysis of the PUR samples with different amounts of DAF is shown in **Figure S18.**

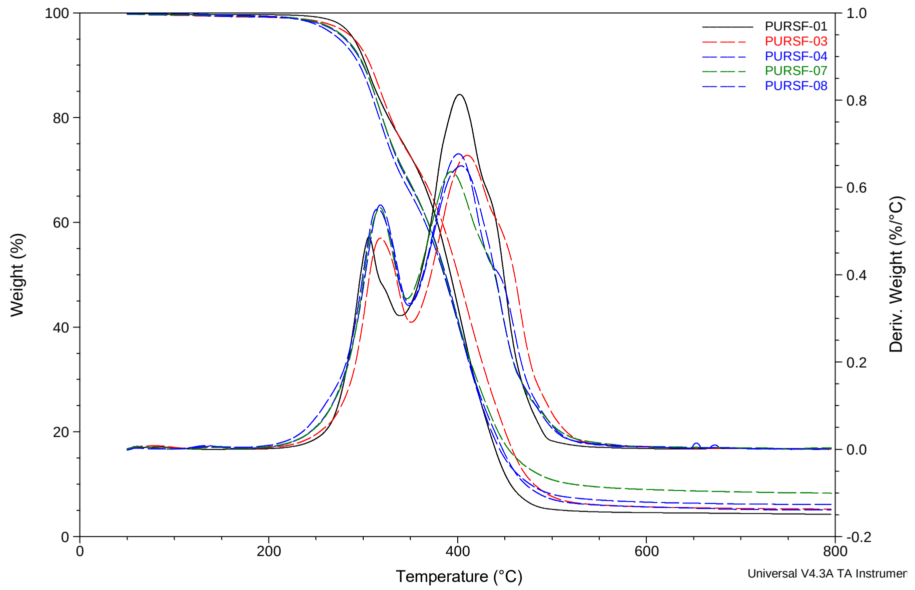

Figure S18. TG and DTG curves under a nitrogen atmosphere at a rate of 10ºC min-1 for the solvent-free adhesive (PURSF-01) and the solvent-free adhesives with DAF (PURSF-03, PURSF-B04, PURSF-07, PURSF-08).

The DSC heating scans for samples with different contents of thermoreversible bonds are displayed in **Figure S19**. It can be clearly seen that except for the control samples PURSF-01 and PURSF-02, all the samples presented a broad endothermic peak ranging from 90 to 150 °C with a maximum at around 132 °C, assigned to the rDA reaction. In addition, the DSC results indicate that the enthalpy associated with the rDA process increases as the content of thermoreversible bonds increases, irrespective of the use of FA in the formulation.

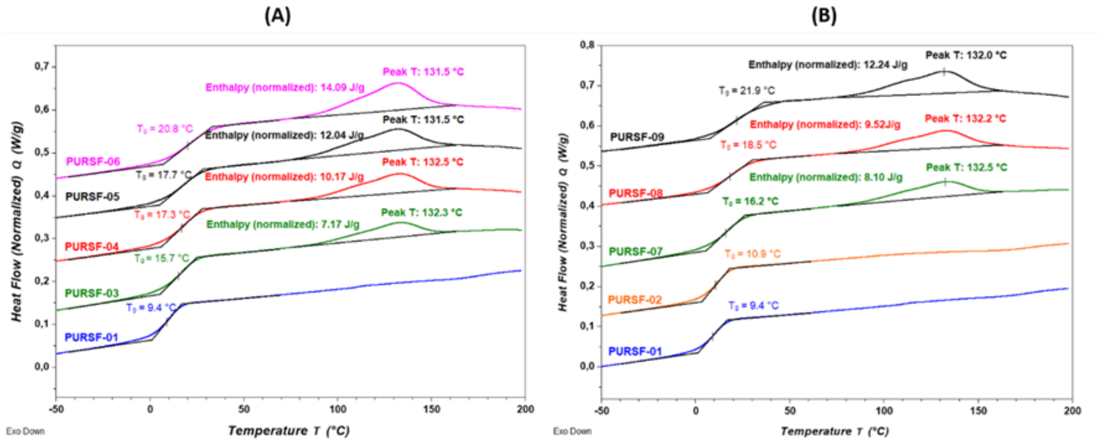

Figure S19. DSC heating curves at 10 °C/min for the solvent-free adhesives prepared in this study. Reference samples without DAF are represented for comparison and samples prepared without and with FA are displayed in (A) and (B), respectively.

3.1. Initial adhesion trials

Hand lamination in the laboratory and pilot-scale or industrial-scale lamination can produce very different results. Indeed, the adhesives employed for lamination are very

different in properties to those used for bulk material adhesion since there is an important optimization process that includes surface pre-processing of the films, adhesive layer thickness, laminating speed, roll pressure, etc. In order to observe whether any major differences could be found between the commercial adhesive and the adhesive formulation including DA adducts, a simple lab-lamination process was devised and a t-peel test was performed to measure their relative adhesive strength. Several laminates of corona-activated low-density polyethylene (LDPE) films and polyethylene terephthalate (PET) films were prepared in the following manner: A sheet of the corona-treated LDPE was covered with a thin layer of the solvent-free adhesive, either with the commercial formulation or its DAF-modified counterpart, by spreading it uniformly using a metal rod. The top PET layer was then added and the laminates were pressed together with a 5 kg weight for 10 minutes. Subsequently, these multilayer laminates were cured at 50 °C for 20 h and then left at RT for the following 6 days. The sheets were then cut into strips 1 cm wide and 6 cm long and standard t-peel tests were conducted. The t-peel tests performed on samples after thermal processing, as described in the manuscript, are presented in Table S.1.

Table S1. Preliminary adhesion strength tests on laminates after thermal treatment.

Laminate	Adhesive	Peel resistance (N/25mm)
PET55μm/ PE60μm	Commercial	2.48
	DAF-modified	1.66
PET30μm/ PE30μm	Commercial	2.30
	DAF-modified	1.45

References.

1. [1] C. Goussé, A. Gandini. Diels-Alder polymerization of difurans with bismaleimides. Polym. Int 1999, 48, 723-31
2. [2] C. Gousse, A. Gandini, P. Hodge. Application of the Diels-Alder reaction to polymers bearing furan moieties. 2. Diels-Alder and retro-Diels-Alder reactions involving furan rings in some styrene sopolymers. Macromolecules 1998, 31, 314-21.
3. [3] O. Ursache, C. Gaina, V. Gaina. Polyurethanes based on thermoreversible networks designed by Diels-Alder reaction. eX-PRESS Polymer Letters 2017, 11, 467-478.
4.
5. [4] Gandini, A.; Coelho, D.; Silvestre, A.J.D. Reversible click chemistry at the service of macromolecular materials. Part 1: Kinetics of the Diels–Alder reaction applied to furan–maleimide model compounds and linear polymerizations. Eur Polym J. 2008, 44(12), 4029-4036.
6. [5] Zhao, J.; Xu, R.; Luo, G.; Wu, J.; Xia, H. A self-healing, re-moldable and biocompatible crosslinked polysiloxane elastomer. J Mater Chem B. 2016, 4(5), 982-989.
7. [6] Fang, Y.; Du, X.; Cheng, X.; Zhou, M.; Du, Z.; Wang, H. Preparation of living and highly stable blended polyurethane emulsions for self-healing films with enhancive toughness and recyclability. Polymer. 2020, 188, 122142.
